# Anticipated barriers and enablers to signing up for a weight management program after receiving an opportunistic referral from a general practitioner

**DOI:** 10.3389/fpubh.2023.1226912

**Published:** 2023-09-21

**Authors:** Chiara Gericke, Sterling Rippy, Danielle D’Lima

**Affiliations:** ^1^Department of Clinical, Educational and Health Psychology, UCL Centre for Behaviour Change, University College London, London, United Kingdom; ^2^Public Health Team, London Borough of Hounslow, London, United Kingdom

**Keywords:** obesity, weight management, tier 2 service, behaviour change, GP referrals, intervention design, lifestyle programme, programme uptake

## Abstract

**Introduction:**

General Practitioners (GP) are advised to opportunistically refer patients with overweight or obesity to a tier 2 weight management program, but few patients sign up after receiving the referral. Signing up to a weight management program is a behaviour, as such, behaviour change interventions are needed to increase sign ups. However, no research has explored the influences on signing up after an opportunistic referral specifically.

**Aim:**

To investigate the influences (i.e., barriers and enablers) on signing up to a tier 2 weight management service after receiving an opportunistic referral from a GP, using a theoretical framework to inform intervention development.

**Method:**

Semi-structured interviews were conducted with 18 residents from the London borough of Hounslow who were eligible for the service. Interview guides were informed by the Theoretical Domains Framework (TDF). Data were analyzed inductively using Reflexive Thematic Analysis and Coding Reliability to identify influences on signing up, before being deductively coded to the TDF and grouped into themes.

**Results:**

Eight theoretical domains were identified as influences on signing up. Fifteen sub-themes were developed and categorized as either a barrier (5), enabler (3), or mixed (7) influence. Beliefs about Consequences was the most frequently reported influence on signing up. Beliefs that were expressed the most often include how effective the program would be, whether the program is needed to lose weight and whether the program would be compatible with their lifestyle. Leveraging Social Influences and changing patient’s Knowledge could address these beliefs and provide a potential route for Behaviour change.

**Discussion:**

The present study provides the first insight into behavioural influences on signing up for a weight management service opportunistically using a validated theoretical framework. This study has implications for intervention development in that public health researchers can identify intervention, content and implementation options based on the findings. Interventions targeting the key domains of Knowledge, Social influences and Beliefs about consequences would likely be the most effective because of their prominence and influence on other domains.

## 1. Introduction

The most recent health survey for England showed that in 2022, around 63.8% of adults in England were living with overweight or obesity (1). It is estimated that if current trends continue, over the next 10 years, the National Health Service (NHS) will spend more than £6.1 billion a year on obesity related illness and more than 2 million quality adjusted life years will be lost (2). The NHS operates a Tiered Care Weight Management Pathway, consisting of four tiers of weight management services for individuals living with overweight or obesity (3). Local and regional public health teams are responsible for providing Tier 1 services, often carried out in a primary care setting, where GPs, nurses and health visitors are responsible for identifying individuals at risk of excessive weight gain and providing advice and resources to help them manage their weight (4). Local authorities are responsible for commissioning Tier 2 services, which are typically 12 weeklong group-based programs that incorporate diet, physical activity and behaviour change components (5). These are also referred to as behavioural weight management services and are also offered commercially (such as Weight Watchers and Slimming World) with the NHS occasionally covering the cost of participation in a commercial program where no community-based program is available. Clinical commissioning groups are responsible for commissioning Tier 3 and Tier 4 services, which are specialist weight management services consisting of surgical and non-surgical treatments such as intensive medical weight management and Bariatric Surgery (6). Since the implementation of the tiered weight management system, numerous studies have found that tier 2 services result in significant weight loss, are more effective and cheaper than primary care-based services led by clinical staff (7–10). As a result, in May 2014, the National Institute for Health and Care Excellence (NICE) published guidelines recommending that primary care physicians refer adults with overweight or obesity (a BMI of at least 25 kg/m^2^) to a tier 2 service to manage their weight in the first instance (11).

The guidelines were published in response to trials which found tier 2 services to be effective at helping people seeking treatment lose weight but until 2016, there were no studies which examined whether a referral to a tier 2 service from a GP to people not seeking treatment would be effective. The Brief Interventions for Weight Loss trial led by researchers from the University of Oxford [BWeL: (12)] was the first to explore whether an opportunistic referral was effective for reducing bodyweight and acceptable to patients. The opportunistic referral took place when patients visited a GP for a routine consultation, the GP noticed that they were above a healthy weight and offered to refer them to a program. Their study published in The Lancet found that of the 722 participants who received the referral, 77% accepted the offer and signed up (13). Of this group, 40% attended the program and after 1 year, had lost an average of 5 kgs, which was 1.43 kg more than the control group who received weight loss advice but was not referred to a program. Eighty-one percent of patients in this study felt that the referral was appropriate and helpful (13). The opportunistic referral that the researchers designed was a brief, 30 second conversation between the patient and the GP, which cost the NHS an average of £22 per patient. A post-hoc analysis determined that most people in the United Kingdom see their GP at least once a year, and that if all patients with overweight or obesity were offered a brief opportunistic referral, it could reduce levels of heart disease in the population by 22% over 10 years (14) and can be a low cost and extremely effective behavioural intervention if the patient joins the program.

However, there is large heterogeneity in the uptake of tier 2 services. A review of the literature between 2000 and 2018 found 26 weight management studies which reported uptake of tier 2 services. The results showed that uptake ranged from less than 1 to 99% depending on the recruitment method (15). Studies that used GP referrals were considered to have ‘medium uptake’, ranging between 21 and 49% (15). Additionally, there are demographic biases in who joins weight management programs. A cross sectional survey of 26,113 adults in the United Kingdom showed that women are significantly more likely to join a weight management program than men and that people from less deprived areas are more likely to join than people from more deprived areas (16). More specifically, a randomized controlled trial as part of the Weight loss Referrals for Adults in Primary care study [WRAP; (17)] found that of 13,949 patients referred to a tier 2 service by a GP, 68% of patients who joined were female, 90% were of white / white British ethnicity, and only 4.6% were below the age of 40 (18). These findings have implications for public health, as low uptake limits the efficacy of weight management programs and demographic biases in who joins these services may contribute to health inequalities.

In order to improve the uptake of tier 2 services, behaviour change is needed because the act of joining a weight management program is a behaviour. Heterogeneity in the uptake of these services is the result of certain factors influencing a patient’s behaviour in that moment. Therefore, it is important to understand what these factors are in order to design effective interventions that address them. The behavioural sciences offer a range of evidence and theory-based approaches to understand influences on behaviour which can feed into the design of interventions to change the behaviour. One such integrated approach is the Behaviour Change Wheel (BCW), which is a synthesis of 19 frameworks of behaviour change found in the literature (19). The BCW has been used by local government and partners such as the NHS and emergency services to guide behaviour change (20) and has been used to explore influences on other weight related behaviours such as healthy eating (21), engaging in physical activity (22), active travel (23), and managing diabetes (24). However, it has not been used to explore influences on joining a tier 2 service. Broadly, the BCW breaks down the method for designing behaviour change interventions into three stages; (1) Understand the behaviour which needs to be changed, (2) Identify intervention options to change the target behaviour, and (3) Identify content and implementation options (25).

The first step in designing effective behavioural interventions is to understand and select the behaviour which needs to be changed (25). Focusing on the behaviour of signing up specifically is a useful starting point, because it is the initial behaviour that will need to take place for patients to subsequently participate in and complete the program. It is also important to specify the target behaviour in appropriate detail and in its context. Evidence has shown that sign-up rates vary depending on whether the patient was referred verbally or via a letter or whether the GP directed them towards more information to sign up themselves or offered to complete the referral for them. For example, in the WRAP randomized controlled trial, 13,949 patients were sent letters of referral from their GP, of which 6.5% signed up (18). In a similar randomized controlled trial 8,810 eligible patients in Birmingham were referred to various weight management programs via a letter from the GP and only 11% signed up (10). In contrast, in the BWel trial, 722 patients were attending a face-to-face appointment in the GP’s clinic when they suggested signing up and offered to complete the referral for them, of which 77% signed up (13). Thus, the behaviour of signing up to the program opportunistically, in the GP’s clinic, after accepting the opportunistic referral, is a promising target behaviour, which would likely have a large impact on the uptake of tier 2 services.

The next step is to identify influences on the target behaviour, using theory-based behavioural science frameworks. A growing body of evidence suggests that interventions developed with an explicit theoretical foundation are more effective than those lacking a theoretical base (26). The Theoretical Domains Framework (TDF) is a validated behaviour change framework related to the BCW which is frequently used to understanding influences on behaviour in clinical practice (25). The TDF was developed by a panel of 32 experts in behaviour change who together identified 128 theoretical constructs from 33 behaviour change theories and simplified them into 14 domains (25). The 14 domains represent cognitive, affective, social, and environmental influences on behaviour with each domain representing several related theoretical constructs (27, 28). Using validated, theory-based behaviour change frameworks such as the TDF ensures that researchers identify and categorize barriers and enablers that will influence the target behaviour, as opposed to identifying influences which might not change the behaviour in practice (27).

Within the related literature, a subset of primary research has explored factors that act as barriers (preventing, discouraging) or enablers (supporting, encouraging) to patients joining lifestyle interventions more generally. Patients report many barriers to joining a program in general, including previous negative experiences, fear of the unknown, a lack of confidence and denial of a problem (15). The wider weight management literature finds that the belief that individuals can lose weight on their own is one of the biggest factors that prevents them from accepting help (29). Practical factors such as scheduling compatibility, cost of the program, time and location of classes also act as barriers to joining a program (30). Patients also report factors that encourage them to join weight management programs such as a desire to improve their health, weight, and self-confidence or receiving results from a medical assessment (15). The referring provider also plays a role in whether patients join a service, as patients may be more likely to join if the referring provider supports their weight autonomy (30).

In 2018, a team of behaviour change experts produced a literature review for Public Health England where they mapped these influences to the TDF. The review found that the most common TDF domains that prevent program uptake are Social influences, Knowledge, and Emotion, which implies that people who do not have social support, have poorer knowledge, and negative emotions are less likely to join these programs. The most common domains that drove program uptake were Intention and Knowledge, which implies that people who intend to sign up and have better knowledge are more likely to join (15). However, none of the studies included in this report, and no research to date, has focused on the behaviour of signing up after an *opportunistic* referral specifically. Instead, the participants in these studies had either been invited to join various programs in writing, via media adverts or sought out a program themselves. Furthermore, many of these programs were not tier 2 weight management programs, but rather lifestyle interventions more generally. Thus, the influences identified to date would not be relevant to this target behaviour and so their application in designing behaviour change interventions is limited. For example, an influence identified in the report “Awareness of programme” (Knowledge) would be an important influence on self-referral to a weight management program but would not be necessary to sign up in the context of an opportunistic GP referral.

It is urgent that we develop a more thorough understanding of what influences the behaviour of signing up to a tier 2 service after being referred by a GP opportunistically. Recently, funding has been made available both from the NHS (31) and Public Health England and National Institute for Health and Care Excellence (32) to expand tier 2 services and cover the cost of GP referrals to these services. Furthermore, guidance has been produced for GPs on how to have the referral conversation with patients (33, 34) based on research (35, 36) from a GP’s perspective, that does not utilize theory or reference frameworks such as the TDF. Developing a thorough understanding of influences on the target behaviour is necessary to ensure that interventions to increase sign-ups are congruent. In the absence of an intervention grounded in evidence-based behaviour change theory, we risk missing out on a large proportion of people who would have otherwise signed up to these services. Thus, this study aimed to use the TDF to understand what influences the behaviour of signing up in this context, to inform future intervention design. The research question that this study aims to answer is:

What are the influences (i.e., barriers and enablers) on signing up to a tier 2 weight management service after receiving an opportunistic referral from a GP?

## 2. Methods

### 2.1. Design

This was a qualitative study which analyzed primary research in the form of semi-structured interviews. Quantitative measures were used in the form of coding reliability. The methods are described in accordance with the consolidated criteria for reporting qualitative research (COREQ) checklist for qualitative research [(37); see Supplementary File 1] and the 15-Point Checklist of Criteria for Good Thematic Analysis [(38); see Supplementary File 2].

### 2.2. Ethical approval

Ethical approval was obtained from the University College London Research Ethics Committee (Project ID: 22079/001). Informed consent was sought via an information sheet and consent form present on the initial survey. All participants gave informed consent to take part in the research.

### 2.3. Case study borough

The London borough of Hounslow was selected as the case study borough for this research. The borough of Hounslow is situated in West London, forming part of outer London and has a population of 272,976 (39). As of 2021, Hounslow had the 7th highest percentage of adults (18+) classified as overweight or obese (56.3%) in London. Within the borough itself, there is a large variability in obesity rates across different wards. Amongst adults, obesity rates are greatest in the Mid-West of the borough, with Heston Central, Hounslow West and Hounslow Heath having the highest rates, and Chiswick wards in the east having the lowest. Previous research from the borough has shown that there is a moderate positive relationship between areas with high levels of deprivation and high levels of obesity (39).

At the time of this study, Hounslow had successfully implemented a tier 1 weight management service and recently secured funding to introduce a tier 2 service. The first cohort of residents had signed up to the tier 2 service and were completing the 12-week program. Data gathered showed that the sign-up rate was low and that residents from the West of the borough, minority ethnic communities and men were under-represented, which reflects the literature more broadly (18).

### 2.4. Recruitment

Promotion started in May 2022. Participants were recruited from the population in Hounslow. Advertisements were created and shared by the borough of Hounslow via newsletters and social media channels. A recruitment team from the borough also handed out physical flyers to passers-by in the street (Supplementary File 3). The advertisement contained a link to a survey, where individuals were required to answer a set of questions to determine whether they met the criteria to participate in the weight management service, and thus, the interviews (Supplementary File 4). To be eligible for the service, individuals need to be (i) a resident in the borough, (ii) between 18 and 65 years old and (iii) have a BMI greater than 25 kg/m^2^ (classified as overweight). Residents are unable to participate in the service if they are (iv) pregnant, (v) suffer from an unmanaged co-morbidity, (vi) have an eating disorder or (vii) have had bariatric surgery in the two years prior. The advertisement also stated that eligible participants would be offered a small incentive (£10 Amazon voucher) to participate in the interviews should they be invited. Promotion ended and the survey stopped accepting responses in June 2022.

**Figure 1:**
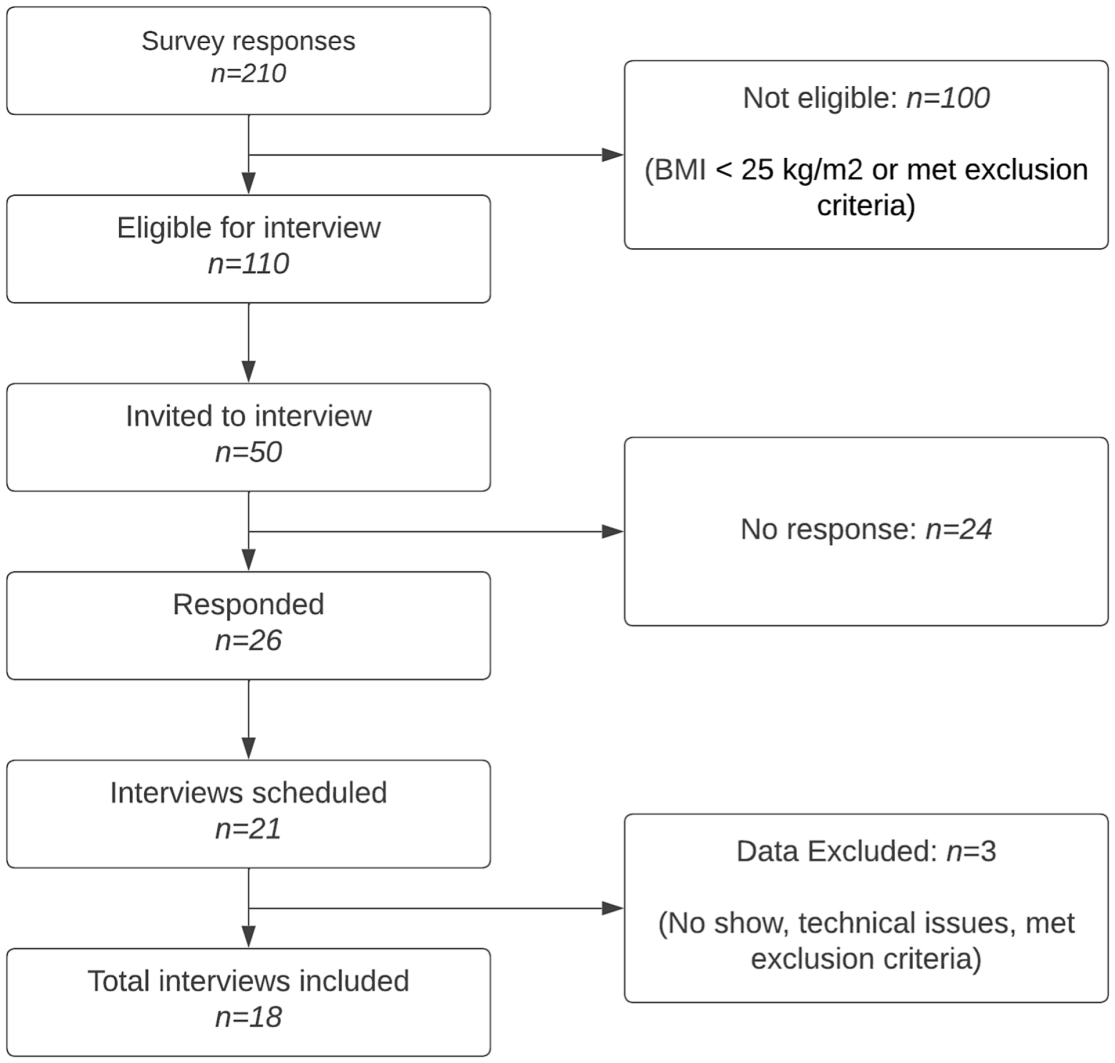
Flow diagram of participant recruitment.

A total of 210 individuals completed the survey, of which 110 met the inclusion and exclusion criteria set out above. The primary researcher (CG) then prioritized 50 residents to be interviewed and contacted them via email to ask if they would be interested in taking part. A maximum-variation (heterogeneity) sampling technique was used to select potential interviewees based on BMI and demographic information (age, sex, and ethnicity). This approach was adopted to recruit individuals who are underrepresented in weight management programs. BMI was calculated by the primary researcher (CG) using individual’s self-reported height, weight, age, and gender on the “NHS Calculate BMI” tool (40). Twenty-six residents replied and expressed interest. Interviews were scheduled with 21 residents, of which one failed to turn up, one was terminated early due to technological issues, and one was excluded from data analysis for meeting an aspect of the exclusion criteria. Figure 1 outlines the process of participant recruitment. All interviews were conducted by the primary researcher (CG).

Recruitment concluded after conducting 18 semi-structured interviews, guided by the concept of information power (41). Information power suggests that a lower number of participants suffices when the sample holds substantial information relevant to the study. In this case, the study’s narrow aim and highly specific target behaviour (19), contributed to the decision. Participants possessed characteristics highly specific to the study aim, as they were all eligible for the service and screened through an extensive survey. Additionally, the researchers utilized established theoretical frameworks to shape the interview guide and data analysis. The quality of dialog was strong, owing to the researchers’ prior interviewing experience and the participants’ willingness to engage. Furthermore, the chosen analysis strategy enables an in-depth examination of narratives and discourse details from a select few participants (27). Thus, the primary researcher (CG) felt that these interviews yielded sufficient information power to meet the study’s objectives. No residents declined to be interviewed or dropped out of the study after agreeing to take part.

### 2.5. Procedure and materials

Semi-structured interviews were conducted in June 2022 via Microsoft Teams or Zoom by the primary researcher (CG). Since community-dwelling adults were recruited via advertisements, not following a recommendation from a GP, participants were emailed a one-page information sheet about the borough of Hounslow’s weight management program before the interview took place (Supplementary File 5). Public health officials from the borough provided details on what to include in the information sheet to reflect the basic information that a GP would share with a patient, so that they understood what they would be committing to if they were to sign up. The one-page information sheet addressed four key points for clarity. Firstly, it provided an overview of what the weight management program entails. Secondly, it outlined the program’s goals. Thirdly, it offered insights into Hounslow’s weight management program structure, including a summarized 12-week timetable. Lastly, it explained the process for signing up, highlighting that GPs frequently make opportunistic referrals for patients during clinic visits. This was presented again by the researcher at the beginning of the interview.

A semi-structured interview guide was developed, informed by the TDF (Supplementary File 6). The TDF served to ensure that relevant influences on behaviour were being considered during the development of the interview guide, as opposed to providing structural specification (42). The TDF was operationalized in such a way that the language was relevant to the target population. For example, the term “sign up” was intentionally selected as the most appropriate and relevant term to describe the target behaviour. While various terms can be associated with enrolment in the program, “signing up” suggests a less formal process, and accurately conveyed the accessible nature of the behaviour. The interview guide contained one open-ended question for each TDF domain to elicit the first response. Broader questions were followed by more detailed prompts to ensure that residents’ responses were fully explored. The interview guide did not consist of questions that related to the domain of Behavioural Regulation, as the target behaviour is a one-off behaviour as opposed to a habit or long-term behaviour to be changed. Questions were structured in a logical order in the interview guide, but the order was used flexibly during the interviews to follow the natural flow of the conversation (27). The interview guide was pilot tested with another researcher from UCL to check comprehension and updated accordingly, before being used with study participants. Table 1 shows an example of five interview questions and the TDF domain that each question relates to.

**Table 1:** Example interview questions.

Interview questions	TDF domain
What do you think the good and bad things about signing up are?	Beliefs about consequences
Compared to your other priorities, how important is signing up to a weight management program?	Goals
Have you ever talked about weight management programs with other people?	Social influences
Can you tell me about the things that you would consider before signing up to the program?	Memory, Attention and Decision Processes
Is there anything that would make you want to sign up for the program?	Reinforcement

Each interview lasted between 30 and 60 min. Fieldnotes were made after each interview to document reflections. The interviews were recorded and transcribed verbatim by a transcription agency. Transcripts were pseudonymized to remove identifiable information and returned to the researcher for analysis.

### 2.6. Analysis

**Figure 2:**
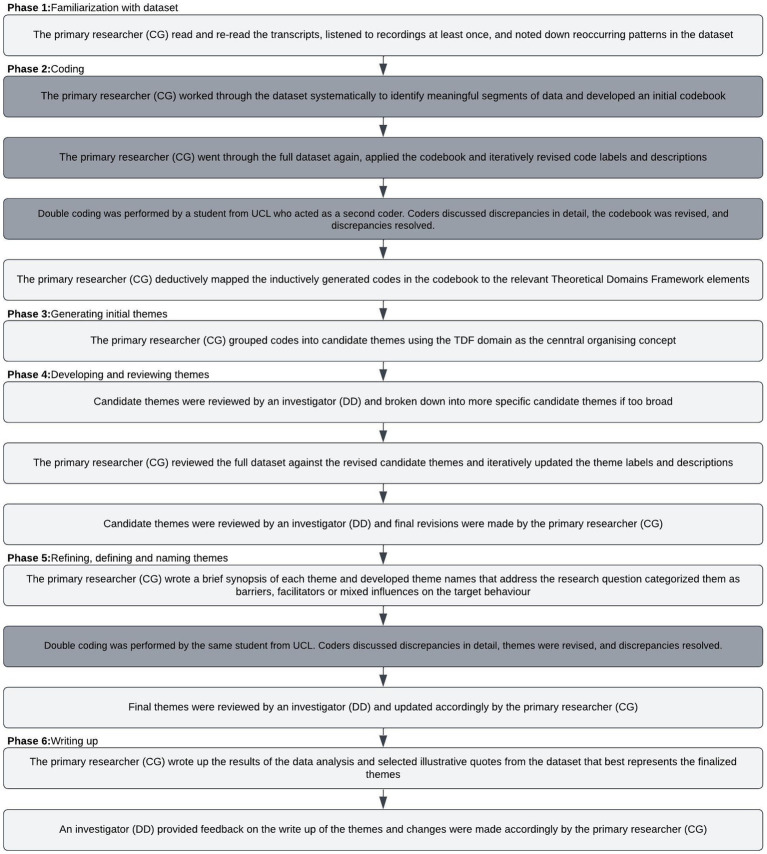
Overview of data analysis approach. Flow diagram showing the steps in the reflexive thematic analysis approach (unshaded boxes) and Coding Reliability approach (shaded boxes).

Data analysis followed Braun and Clarke’s six phases of Reflexive Thematic Analysis (38) but incorporated aspects of the process of Coding Reliability (43). Figure 2 summarizes the key steps taken and illustrates which elements of Reflexive Thematic Analysis and Coding Reliability were used.

Reflexive thematic analysis was chosen to analyze the data because the method is flexible with regards to data collection, dataset size, analytic strategy (inductive – deductive analysis), and allows for the incorporation of relevant theory to inform data analysis. Given that the aim of this research was to develop a thorough understanding of the target behaviour, the data was initially coded using an inductive, ‘bottom up’ approach, in which there was no attempt to fit the data to existing theory. Adopting this approach facilitated an understanding of different experiences, perspectives and meaning in a topic area that has not been widely explored in the past. This approach is in line with guidance from McGowan et al. (42) for assessing behavioural influences in qualitative studies.

Considering that the findings may be used to inform intervention design, it was decided from the outset that coding would be performed at the semantic (i.e., participant driven, descriptive) level. Quality was conceptualized as accuracy and reliability of observations, and interrater reliability would be used to measure this. Thus, the research was underpinned by a realist ontology (44), which gave rise to a post-positivism epistemological approach (45). After an interrater reliability check had been performed with the inductively generated codebook, the codes were deductively coded to the relevant TDF domains. This made it possible to identify codes (i.e., influences) which were not relevant to the behaviour of interest. This step was necessary because as described above, much of the related literature has explored barriers and enablers to joining a program more generally, which might not change behaviour in practice.

After the inductively generated codes were deductively coded to the TDF, the TDF domains were used as the central organizing concept to group the data into candidate themes. A second interrater reliability check was performed to arrive at a consensus of what the candidate themes represent. After a satisfactory agreement had been reached, candidate themes were finalized and labeled as sub-themes. The sub-themes reflect a shared topic and aim to capture the diversity of meaning in relation to the target behaviour. Sub-theme names were developed which can be interpreted as domain summaries (46). The TDF domains formed the main themes. Adopting this approach was informed by the aim of understanding the influences on the target behaviour using a theory-based framework.

Finally, following guidance from Braun and Clarke (47) on theme development, a smaller number of themes were prioritized to ensure that each could be explored fully and to avoid having a greater number of thin or underdeveloped themes. The primary researcher (CG) prioritized themes based on their frequency across the interviews.

### 2.7. Coding reliability

To ensure the reliability of the coding process, double coding was performed at both the inductive and deductive stages of data analysis (Table 2). At the inductive stage, a researcher from UCL double coded 20% of the transcripts using the initial codebook developed by the primary researcher. An interrater reliability check revealed a Cohen’s Kappa of 0.65, suggesting that moderate reliability had been achieved (48). The two coders discussed the discrepancies in detail, the coding frame was revised, and discrepancies resolved. A second round of double coding was performed to assess the reliability of the deductive coding to the TDF. The same second coder coded 100% of the final themes to the TDF, which revealed a Cohen’s Kappa of 0.66, indicating moderate reliability (48). The discrepancies were discussed, resolved and the coding frame updated accordingly. All coding and double coding was performed using the software package Nvivo 2020.

**Table 2:** Calculation of interrater reliability (IRR).

	Percentage of double coded data	Average Cohen’s Kappa	Percentage agreement (%)
Inductive coding	20%	0.65	99.49
Deductive coding	100%	0.66	83.33

Interpretation of Cohen’s kappa is based on McHugh (48) (value of kappa – level of agreement: 0.0–0.20 – none; 0.21–0.39 – minimal; 0.40–0.59 – weak; 0.60–0.79 – moderate; 0.80–0.90 – strong; above 0.90 – almost perfect).

Although data analysis is described in linear terms, the process of the initial codebook development was iterative, and codes were further refined or broken down into a smaller number of codes if it was evident that they reflected more than one TDF domain.

## 3. Results

A total of 18 residents participated in this research. Table 3 shows participants by gender, age, ethnicity, and BMI.

**Table 3:** Participants by gender, age, ethnicity, and BMI.

	Men (*n* = 9)	Women (*n* = 9)	Total (*n* = 18)
*BMI*			
25 kg/m^2^ ≤ BMI < 30 kg/m^2^ (overweight)	5	4	9
30 kg/m^2^ ≤ BMI ≤ 63.9 kg/m^2^ (obese)	4	5	9
*Age*			
18–24	0	1	1
25–34	3	3	6
35–44	3	2	5
45–54	2	2	4
55–64	1	1	2
*Ethnicity*			
White British or other White background	4	3	7
Asian or Asian British	4	3	7
Black, Black British, Caribbean or African	0	3	3
Mixed or multiple ethnic groups	1	0	1

Eight themes and 15 sub-themes were developed from the semi-structured interviews. Each sub-theme was categorized as either a barrier (5), enabler (3) or mixed (7) influence. Mixed influences acted as a barrier for some but an enabler for others. Table 4 in Supplementary material presents a summary of the results, frequency of the sub-themes (based on total number of interviews, 18 maximum) and additional illustrative quotes.

### 3.1. Beliefs about consequences

#### 3.1.1. Efficacy of the program

Participants held beliefs about whether the program would be effective at helping them lose weight and improve their health if they did sign up. Some participants believed that the program would produce many of the intended health, wellbeing, and weight loss benefits, which acted as an enabler to signing up:

*“… the good things is reducing weight, and getting the knowledge and the understanding of healthy eating exercise programmes, what to do, and the benefits of it all” (Male, 35–44, White British or other White background, with obesity).*

In contrast, other participants believed that the program would not successfully help them lose weight, which acted as a barrier to signing up:

*“Again, I’m not quite sure how effective it would be” (Male, 55–64, White British or other White background, with overweight).*

Tied to this, these participants often expressed a preference for other weight loss programs or strategies that they believed would produce more effective results than this program.

The sub-themes “Understanding benefit of the program” and “Others experience with weight management programs” are related in that these influenced participants belief about the efficacy of the program.

#### 3.1.2. Need for a program

Participants held beliefs about whether they would need the support of the program to lose weight if they did sign up. One of the biggest barriers to participants signing up was the belief that they can manage their weight independently without the support of a program:

*“For me it’s not a case of something is deficient or anything else, it’s just bad habits that I do get through. Like I do not require intervention” (Female, 25–34, Asian or Asian British, with obesity).*

They often described that they already know what is contributing to their weight gain and the steps that they need to follow to lose it. In contrast, other participants described that they struggle to manage their weight independently, and do need the support of a program to lose weight:

*“Of course, every time I tried to lose weight on my own or manage my own weight … the opposite would happen … I realized that I’m not able on my own to maintain a healthy, to maintain my weight for long” (Female, 45–54, Black, Black British, Caribbean or African, with overweight).*

The sub-theme “Trust in GP and their recommendation” is related in that participants were more likely to believe that the program was necessary if they trusted their GP.

#### 3.1.3. Compatibility with lifestyle

Participants held beliefs about whether the program would be compatible with their lifestyle and existing schedules if they did sign up. The times, dates and locations of the classes were often described as factors that made the program compatible or incompatible. Some participants described that the program would not be compatible, because they had other work or childcare responsibilities, which acted as a barrier to signing up:

*“Some of the classes are physically going to the place and I would not be able to do that personally because I do not, I would not be able to find childcare for the times that they wanted you to” (Female, 18–24, White British or other White background, with obesity).*

Other participants described that the times, dates, or locations of the classes would work well for them, which acted as an enabler to signing up:

*“I do travel around Hounslow anyway, I live in Feltham so it’s not a problem” (Male, 45–54, White British or other White background, with overweight).*

#### 3.1.4. Negative emotional response to participation

Participants believed that they would experience negative emotions such as stress or anxiety if they were to participate in the program, which acted as a barrier to signing up. Participants described that they were worried about being judged or feelings of shame because of their weight:

“I think it’s just because it’s a shame thing, I’m not very active, that kind of thing, so nobody needs to see me at my worst” (Female, 25–34, Asian or Asian British, with obesity).

Other participants believed that being on the program would trigger their anxiety:

*“So there’s obviously the flipside of whilst the accountability’s good, it can also be negative because it can trigger my anxiety if I’ve not been able to do it, for whatever reason” (Female, 25–34, White British or other White background, with obesity).*

Participants also expressed worries in relation to procedures that would need to take place on the program, such as feeling uncomfortable if someone were to measure their weight in front of others.

### 3.2. Knowledge

#### 3.2.1. Having practical information

Having practical information about the program (such as times, dates and locations of classes) acted as an enabler to signing up whilst in the GP clinic. If this information was provided to participants, it would enable them to sign up in the moment:

*“I plan within the next couple of days what I’m going to do. So, if I’ve got that timetable in front of me and I can go, “OK, I can meet at that time”” (Male, 35–44, White British or other White background, with obesity).*

Participants described that they needed this information to determine whether it would be feasible for them to accept the referral because they had work, childcare or other responsibilities to consider:

*“I would just need more details, as someone who works shifts, about the timing of it” (Male, 35–44, Asian or Asian British, with obesity).*

#### 3.2.2. Misconceptions of who weight management programs are for

Participants held misconceptions about who weight management programs are meant for, which acted as a barrier to signing up. A common misconception was that weight management programs are only meant for people with obesity, and not overweight:

*“Is it anyone who’s, even if you are five kilos overweight, you can do it? I do not know. I do not know. My understanding was that you’d have to be quite overweight” (Female, 45–54, White British or other White background, with overweight).*

Other common misconceptions were that weight management programs are specifically meant for people who are immobile, old, retired, have mental illnesses or substance abuse issues. Participants also thought that weight management programs are meant for people who have medical issues, such as diabetes or high blood pressure, since the program is recommended by the GP:

*“if it’s through your GP, I … would assume like it’s a, you know, solving a medical issue rather than promoting like a healthy lifestyle” (Male, 25–34, White British or other White background, with overweight).*

The sub-theme “Fit for the program” is closely related in that people’s misconceptions determined whether they felt like a good match for the program.

#### 3.2.3. Understanding benefits of the program

Understanding how the weight management program would improve their health and weight acted as an enabler to signing up. Participants described that they wanted to understand from the GP how the program would benefit them:

*“I want to talk about with the GP to say “why are you recommending the programme? What do you think I’ll get out of it?”” (Male, 35–44, Asian or Asian British, with obesity).*

If they understood how the program would help them or fill a gap that they were missing, they were more likely to sign up. Many participants described that they would not sign up if they did not understand how the program would benefit them, because they did not want to feel like they were only being referred to tick a box:

*“I would assume that there is a reason that they are offering it and [if] I am convinced that it’s not just a tick box. I am probably, like 95% likely to sign up” (Female, 35–44, Black, Black British, Caribbean or African, with overweight).*

### 3.3. Goals

#### 3.3.1. Importance of weight loss

How important weight loss is to participants influences whether they sign up to the program. If weight loss was important to them, this acted as an enabler to signing up. Certain factors made weight loss more important, such as having suffered from weight related health problems in the past:

*“They said to me that I was diabetic and it almost killed me. Now I’m pre-diabetic. For me, it’s top on the list” (Female, 45–54, Black, Black British, Caribbean or African, with overweight).*

Some participants also described that their ethnicity puts them at risk of developing weight related illness, which makes it more important for them to be on a program.

In contrast, other participants described that their weight was not a concern or a priority for them which acted as a barrier to signing up. They often described other priorities which were more important to them such as work, exams, or family obligations. These participants described that they would not sign up to the program because they need to focus their time and attention elsewhere:

*“So I’ve started a new role with the same employer, that’s my priority at the moment, exercising is not. Weight management is not” (Female, 45–54, White British or other White background, with overweight).*

Some participants described that their current weight determines how important it is to lose weight and that if they were to become heavier, weight loss would be considered more important.

### 3.4. Social influences

#### 3.4.1. Trust in the GP and their recommendation

Participant’s trust in the GP and their recommendations, or lack thereof, influences whether they sign up. Some participants described that they do not trust what the GP recommends, which acts as a barrier to signing up:

*“I do not know because, mm, I feel as if, like I said before, there’s always that issue where I cannot always trust his view” (Male, 25–34, Asian or Asian British, with obesity).*

Not trusting the GP was often linked to previous negative experiences with healthcare professionals. In contrast, other participants described that they do trust the GP, which acts as an enabler to signing up:

*“It’s important to listen to the professionals regardless of what I’m seeing, what I feel myself. I think it’s important” (Female, 45–54, Black, Black British, Caribbean or African, with overweight).*

Some participants described that there is one healthcare professional in particular that they have a good relationship with or who knows their medical history well that they would likely listen to if they made the referral:

*“If they know you then you trust them more. So, you know, if my actual proper GP who would know me versus a locum, if my actual, proper GP recommended it, then I would pay more attention” (Female, 35–44, Asian or Asian British, with obesity).*

This sub-theme is closely linked to the sub-theme “Need for the program”, in that participants who trusted the GP’s recommendation were more likely to believe that the program was necessary for them:

*“I would definitely be taking their advice on stuff because they are right, at the end of the day, you know. I know that my weight at the moment is not great and I need to do something about it … So if they suggested something that could be done … they know better” (Female, 25–34, White British or other White background, with obesity).*

#### 3.4.2. Other experience with weight management programs

Many participants knew of others who had been on a similar weight management program in the past. Witnessing their weight loss journey impacted how likely they were to sign up to this program. Participants had often heard negative feedback from others about similar programs, or had noticed that as soon as the program had ended, others had put the weight back on again:

*“I’ve known a lot of people who have done these sort of … programmes and I’ve never known anyone to keep it off” (Female, 25–34, White British or other White background, with obesity).*

This acted as a barrier to signing up. In contrast, other participants described that they know people who have had a very positive experiences on similar programs, which encourages them to try it for themselves:

*“I saw results from that, from a friend as well. So, then I was like “Oh damn, that works.” And they have got results that I wanted, so yeah, absolutely” (Female, 25–34, Asian or Asian British, with obesity).*

### 3.5. Social/professional role and identity

#### 3.5.1. Fit for the program

Whether participants feel like a good fit for the program influences sign up. This sub-theme is closely related to “Misconceptions of who weight management programs” are for as participants knowledge of who joins these programs ultimately led them to feel like a match for the program (or not):

*“I suppose I would feel a bit awkward being there, if I do not like have – if I’m not at like high risk of like a heart attack” (Male, 25–34, White British or other White background, with overweight).*

Participants who did not hold misconceptions were more likely to feel like they are the type of person who would be on the program, which acted as an enabler to signing up:

*“Yeah, I am the type of the person that would sign up to it, definitely.” (Female, 25–34, White British or other White background, with obesity).*

Part of what makes participants feel like a good fit for the program is being in a group with “*like minded others*” (Female, 45–54, White British or other White background, with overweight). Many younger participants described that being in a class with older people would make them feel uncomfortable, and similarly older participants described they would feel out of place if they were in a class with only young people. Participants also discussed this from a cultural perspective. Women who wear coverings described that they would not be the right match for a program where men were also participating, because it would be uncomfortable and impractical to cover whilst exercising.

### 3.6. Emotion

#### 3.6.1. Negative emotional response to signing up

Participants described negative emotions that they would experience in the GPs clinic, which acts as a barrier to signing up. Some participants described that receiving the referral would be offensive or make them feel disappointed in themselves. Other participants described that they would feel worried that something was wrong with them:

*“I’d feel worried and concerned that there was something wrong with me, physically, that I needed to go and do this” (Female, 45–54, White British or other White background, with overweight).*

Participants also described that they imagine signing up to the program would make them feel stressed or trigger anxiety:

*“… it would be like a thought in the back of my mind where it’s like a do you really want to put yourself through the stress and the anxiety of doing that” (Female, 18–24, White British or other White background, with obesity).*

### 3.7. Environmental context and resources

#### 3.7.1. Financial implications of joining a program

Participants described that the financial implications of joining a program would prevent them from signing up. They described that despite the program itself being free, they would still find it challenging to cover the associated costs, such as having to buy gym clothing, pay for parking, petrol, or childcare:

*“Also the cost of petrol. That sounds really petty but that’s not petty anymore, you know, why am I going to waste my petrol driving to an exercise class if I can do it online” (Female, 45–54, White British or other White background, with overweight).*

Participants who were in full time employment described that they would need support from their employer to be able to sign up, mostly in the form of paid time off to be able to attend the various classes:

*“If it falls within my working day, my employer has to support me 100 percent” (Female, 55–64, Black, Black British, Caribbean or African, with obesity).*

#### 3.7.2. Needing time to think, plan, and discuss

Needing time to think, plan and discuss acted as a barrier to participants signing up in the moment. Participants described that they would need to be given time to read and process information:

*“I think I would not make a decision right there and then, I would definitely say, you know, can I have all the information first and then think about it and then sign up” (Female, 35–44, Asian or Asian British, with obesity).*

Other participants described that they would want to sit with the GP and talk through the particulars of the program. In some cases, this was perceived to be problematic because of the short appointment time that patients have with their GP:

*“… you have not got the time, in ten minutes, to… because if the GP said “have you thought about this?” then he’d need to tell me when is it, when does it start, there’s no cost in it, you have to have this much time and I want to know when’s it start, where do I have to go and so I’d have all the questions” (Male, 35–44, Asian or Asian British, with obesity*).

### 3.8. Beliefs about capability

#### 3.8.1. Readiness for change

Being ready to join a weight management program influenced participants perceived capability to sign up. Participants described that being in a good place in their lives allows them to focus on their health and bettering themselves, which acted as an enabler to signing up:

*“I would need to be in a good place. Like at the moment, I’m doing reasonably OK so I’m in the place where I could focus on that. Because when I’m not in the best mindset … it’s just more about surviving when I’m in that mode than it is about bettering” (Female, 25–34, White British or other White background, with obesity).*

They described that signing up to a program and starting the weight loss journey is the hardest part of the process, and so it was something that they needed to be in a good place to be able to do:

*“… that’s probably the biggest one is, is this the right time for me? Am I ready, in my life, to do something like this? … because if you are not, what’s the point?” (Female, 45–54, White British or other White background, with overweight).*

## 4. Discussion

The present study provides the first comprehensive assessment of influences on the behaviour of signing up to a weight management service opportunistically, drawing upon a theoretical framework that can inform intervention development. Due to the opportunistic nature of the target behaviour, many barriers to patients accepting weight management support from a GP in the related literature did not influence behaviour in this context. Instead, this study found that the domain of Beliefs about consequences was the most frequently reported influence on signing up. Whether Beliefs about consequences acted as a barrier or enabler was largely driven by the domains of Social influences and Knowledge, providing a potential route for behaviour change. Other domains that influenced the target behaviour include Goals, Emotion, Environmental context and resources and Beliefs about capability. The findings from this study make several novel contributions to the science, and are discussed in more detail below, in the context of the related literature. The findings have theoretical, practical, and clinical implications. First and foremost, the findings can be used to develop targeted interventions aimed at increasing the number of patients living with overweight or obesity who agree to join a tier 2 service after receiving a referral.

Participants hold many beliefs about what would happen after signing up to the program, which influences whether they ultimately would. Beliefs that were expressed the most often include how effective the program would be, whether the program is needed to lose weight and whether the program would be compatible with their lifestyle. The finding that Beliefs about consequences is the most influential domain on the behaviour of signing up is aligned with the health behaviour change literature in general. Conceptual frameworks underlying models of health behaviour such as the health belief model (49), protection motivation theory (50), social cognitive theory (51), theory of planned behaviour (52), theory of reasoned action (53) and transtheoretical model of behaviour change (54) are unified in that they recognize that the decision to adopt a new behaviour is based on an analysis of the costs and benefits associated with different courses of action (55). Many of these models were incorporated in the development of the TDF (56) and so this cost–benefit analysis can be likened to the Beliefs about consequences domain in this context. These theoretical models have been used to inform and analyze vast amounts of research in the weight management literature and have led researchers to uncover different sets of beliefs that are associated with behaviour change (55). For example, using the health belief model (49) to inform data analysis, a similar qualitative study found that individuals’ decisions to initiate weight loss interventions in general were influenced by their perceptions of how effective it would be in helping them lose weight (30). As this study is the first to explore opportunistic sign-ups using a theoretical framework, the finding that Beliefs about Consequences strongly influences sign-ups is both novel and of theoretical importance. However, changing patients’ beliefs on the effectiveness or necessity of a weight management program can be difficult due to factors such as pre-existing attitudes, emotions, and personal experiences. Patients may also not voice their beliefs to begin with, making it challenging to address through an intervention.

The results pose a potential solution to this challenge, in that they find that the domains of Social influences and Knowledge largely influence whether Beliefs about consequences act as a barrier or enabler to signing up. Thus, changing patients’ knowledge, and leveraging social influences, may be two ways to lead them to hold positive beliefs about the consequences of signing up. The links between these domains emerged in various ways in the data. Firstly, this study found that the domain of Social influences influenced Beliefs about consequences in that participants described that if they trust the GP providing the referral, they are more likely to believe that the program is needed and subsequently sign up. Furthermore, they described that there are certain healthcare professionals that they trust more than others and would be more likely to sign up if the referral came from them. Unfortunately, many participants in this study also described that they have had negative experiences with healthcare professionals in the past, who have made them feel at blame for having excess weight. This weight stigma is still pertinent in the healthcare system (57) and may lead patients to hold negative beliefs about the program and decline an offer of weight loss support. These findings are aligned with existing literature, which has found that weight bias from healthcare professionals can lead to an avoidance or delay in seeking medical care and worse health outcomes (57), and is paramount to consider when designing an intervention targeting opportunistic sign ups. Secondly, the domain of Knowledge influenced Beliefs about consequences in that the study found that if individuals understood how the program would benefit them, they were more likely to believe that the program would be effective. These findings highlight the importance of effectively communicating the intended benefits of the program to patients who may not have actively sought treatment. Ultimately, the study suggests that leveraging social influences and improving patient knowledge would likely be effective in influencing beliefs about the consequences of signing up.

Another barrier that many participants reported was feeling like they were not the right fit for the program, which reflects the domain of Social/Professional Role and Identity. Similar to changing beliefs about consequences, addressing patients’ sense of belonging and fitting in with others in the program can be challenging. However, the study identifies a potential solution, as the domain of Knowledge was found to influence Social/Professional Role and Identity. In general, participants were unsure who the weight management service was intended for, and were unaware that individuals with overweight could join the program. Many held the belief that the program was only intended for individuals with obesity, illness, or pre-existing medical conditions. Upon reviewing the one-pager provided (Supplementary File 5), participants were surprised to learn that behavioural weight management programs take a holistic approach to weight management and do not solely focus on the basics of weight loss. These commonly held misconceptions act as a barrier to signing up. Ultimately, a lack of knowledge regarding who can join weight management programs and what they teach could be contributing to demographic biases in who joins the programs (16).

Goals was another highly influential domain on the behaviour of signing up. All participants described that signing up would largely be influenced by how important weight loss is to them. Similar findings have been reported in the related literature (15). Participants reported that certain factors made weight loss less of a priority, such as having competing health, family, or work obligations. Many participants also described that they did not feel that weight loss was important to them at the time of the interview, but that if they were to gain more weight, to the point where they felt it was out of their control, weight loss would be considered more important. In contrast, participants who felt that weight loss was a top priority were more likely to sign up.

This study found that the domain of Environmental context and resources acted as a barrier to signing up, in ways that are both in line with and contribute to the related literature. An intervention targeting opportunistic sign ups overcomes many of the physical barriers to patients receiving weight management support from a GP (such as transportation and the density of GP practices) since the behaviour would occur whilst already in the GP’s clinic. As such, these influences were not relevant in this context but are still barriers (58) which largely limit who is able to be referred to a program and are an important consideration (59). In this study, the domain of Environmental context and resources acted as a barrier to signing up through needing time to think, plan and discuss before signing up. Research conducted with GPs reports similar findings in that GPs feel that they do not have enough time to discuss weight with patients during an appointment as well as address the primary reason for the visit (60). These findings suggest that patients might need additional time outside of the clinic to think about and digest materials before signing up. With regards to findings in line with the related literature, previous research has described that intervention entry is facilitated by having the ability to afford an intervention (30). Despite this weight management program being free, the same principle applied in that participants described that not having the funds to cover the associated costs (such as petrol and parking costs) acts as a barrier to signing up to the program.

Finally, the domains of Beliefs about capability and Emotion were also found to influence sign up, although amongst fewer participants. Similar to the physical barriers described above, an intervention targeting opportunistic sign ups overcomes various Beliefs about capability barriers such as believing that they would be unable to log on to a website or travel to the service to sign up, since the GP would complete the referral for them. Accordingly, participants in this study did not doubt their physical capability to sign up but were concerned about their psychological capability. This emerged through the need to be ready for change to sign up. Participants described that having strong mental health and being in a good place in their lives would make it easier for them to sign up, because they felt this would help them manage the difficulties associated with being on a program. Emotion also acted as a barrier to signing up. Participants reported negative emotions that they might experience in response to hearing the GPs recommendation, such as anger, frustration, anxiety or feelings of guilt. The study conducted by Aveyard et al. (13) found that patients would not be offended or react negatively to a GP discussing their weight with them or referring them for weight management support. This finding has informed many guidelines for GPs in the United Kingdom (61) and is frequently referenced in webinars and reports (62) however the present study found that individuals may indeed experience a negative emotional reaction to a GP telling them that they should consider interventions to manage their weight. GPs should be cognisant of this, be supportive, encouraging and use the correct, non-stigmatizing language when discussing weight with patients (57, 63).

### 4.1. Strengths and limitations

These findings should be interpreted considering several strengths and limitations of the research. A strength of this study was the use of a comprehensive and validated behaviour change framework (the TDF) to understand influences on the target behaviour. Using the TDF allows these findings to be used to inform intervention design following the steps of the Behaviour Change Wheel (25). A further strength of this research is that the researchers replicated the process that a GP would follow to identify individuals who they would refer to the service by adopting the same inclusion and exclusion criteria that the service uses. The primary researcher spoke to these individuals before a GP had spoken with patients, and so their responses can be considered *a priori* accounts of what would influence sign up in this context. However, this is also a limitation in that the participants in this study did not communicate with or verbally receive a referral from a GP. As described above, research has shown that talking about weight with patients is a highly sensitive topic, and that using the incorrect terminology, having a negative attitude, and having stigmatizing material on display (such as magazines or posters) can lead to an avoidance or delay in seeking medical care and worsen health outcomes (57). Thus, patients may have reported additional barriers to those identified in this research had they been referred to the service prior to the study. Height and weight data was also self-reported which could introduce self-report bias. A further limitation of the study is that the sample only consisted of residents from the London borough of Hounslow and so the findings presented may not be reflective of barriers that individuals in other geographical locations may face. However, a strength of this research was the use of a maximum-variation (heterogeneity) sampling method to recruit a diverse sample, including individuals who are traditionally under-represented in weight management programs, which improves the transferability of the findings (64).

### 4.2. Implications

This study has implications for the systematic development of behaviour change interventions targeting opportunistic sign ups to weight management programs. It finds that eight TDF domains are influential on whether participants sign up to weight management programs after receiving an opportunistic referral from a GP. Following the stages of the BCW, a valuable next step would be to identify intervention options followed by content and implementation options based on the findings (25). These findings should be considered as part of a systematic intervention development process, however an illustration of how an intervention might target opportunistic sign ups is described below based on the key barriers and enablers identified in this study.

In order to increase sign ups, interventions targeting the domain of Beliefs about consequences would likely be the most effective and could do so via the domains of Knowledge and Social influences. Focusing on the domain of Knowledge, the Theories and Techniques tool (65) can be used to identify Behaviour Change Techniques (BCTs) that have been shown to link to the domain of Knowledge, to improve patient’s understanding benefits of the program (sub-theme “3.2.3 Understanding benefits of the program”). For example, Information about Health Consequences could be operationalized by providing information on the health benefits associated with being on the program, such as increased weight loss and improved cardiovascular function. Several policy categories could be used to deliver this intervention function such as communications, marketing, or guidelines (25). An infographic or flyer could be designed for GPs to give to patients whilst they are having the referral conversation. This is one example of an intervention component that would likely influence patients’ beliefs about the efficacy of the program (sub-theme #3.1.1) and could form part of an intervention package targeting the remaining barriers and enablers identified in this study. Once we have a clearer understanding of which intervention components address the target behaviour, concrete guidelines should be developed to highlight what is expected of key stakeholders as part of the intervention.

The results also have clinical implications in that they may help GPs to develop a better understanding of the information that patients will be looking for before signing up and what their thought process may be. Public Health England (34) recommends that GPs “become familiar with [their] local services” but do not specify which aspects of the service delivery are important to be familiar with. This study finds that participants require practical information about the structure and timing of the program, who else would likely be participating, the health benefits and learnings that they would gain from being on the program. Having this information available might facilitate a sign up in the moment, as opposed to directing patients elsewhere to find this information. Furthermore, GPs can anticipate that individuals would likely consider whether they need the support of a program, how effective it would be and how important weight loss is to them before deciding to sign up. Addressing these factors in the initial conversation or offering to talk though these points with the patient to make an informed decision may also help facilitate a sign up, as opposed to leaving the patient to ruminate on these factors alone.

Finally, these findings have theoretical implications and extend the science in this area. Guidance has been produced for GPs on how to have the referral conversation with patients (33, 34) based on research (36) that does not explicitly reference theory. However, a growing body of evidence suggests that interventions developed with an explicit theoretical foundation are more effective than those lacking a theoretical base (26). By using a theoretical framework to collect empirical data, evidence of effectiveness can be accumulated across different contexts and populations, which can then inform intervention development and aid understanding of why an intervention is effective or ineffective (26). In the absence of this research, it cannot be said that the guidance produced to date by Public Health England will lead to increased sign-ups or address the barriers that patients face. These results provide the first insight into the behavioural influences on signing up for a weight management service opportunistically using a validated theoretical framework.

### 4.3. Directions for future research

Future research should aim to develop an understanding of influences on subsequent behaviours that are necessary for patients to utilize weight management programs. This study explored influences on the behaviour of signing up to a weight management program but signing up does not guarantee that patients will attend or complete the program. This is evident in the research conducted by Aveyard et al. (13) where only 40% of those who signed up to the program ended up attending. Considering again the Behaviour Change Wheel approach, attending the program after signing up is a different behaviour, which is subject to a range of other influences. In order to design effective interventions to increase attendance, the behaviour of attending after signing up should be explored using the same or similar methodological approach adopted in this study (25). Additional behaviours also need to be better understood to increase the utilization of weight management programs. Numerous studies in the weight management literature have found that there are inequalities with regards to who is offered this service. Similar to participation bias, research has shown that GPs are more likely to refer middle aged, high socioeconomic status (SES), white/white British women to weight management programs (18). Furthermore, research has shown that GPs experience barriers to referring a patient opportunistically such as concern about offending the patient, limited referral pathways and a lack of confidence (60) which also need to be addressed. Thus, this research can be considered the first step along the journey of designing effective behavioural interventions to increase the utilization of weight management programs.

## 5. Conclusion

In conclusion, an intervention focused on increasing the number of patients who sign up for a tier 2 service after receiving an opportunistic referral from a GP has the potential to have a significant impact on the uptake of tier 2 services. This study aimed to explore the factors that influence this target behaviour and is the first to do so using a theory-based behavioural science framework, the TDF. Accordingly, the results indicate that patients’ internal beliefs about the consequences of signing up pose the biggest barrier to doing so. Public health researchers can use the results mapped to the relevant TDF domains to identify intervention options and develop effective content and implementation strategies by following the stages of the Behaviour Change Wheel. The findings from this study suggest that interventions targeting the key domains of Knowledge, Social influences, and Beliefs about consequences are likely to be the most effective at increasing the number of patients who sign up to a tier 2 service opportunistically due to their prominence and influence on other domains.

## Data availability statement

The datasets presented in this article are not readily available due to the sensitive nature of the data, data sharing was not requested or granted from the Research Ethics Committee. Requests to access the datasets should be directed to chiara.gericke.20@ucl.ac.uk.

## Ethics statement

The studies involving humans were approved by Ethical approval was obtained from the University College London Research Ethics Committee (Project ID: 22079/001). Informed consent was sought via an information sheet and consent form present on the initial survey. All participants gave informed consent to take part in the research. The studies were conducted in accordance with the local legislation and institutional requirements. The participants provided their written informed consent to participate in this study. Written informed consent was obtained from the individual(s) for the publication of any potentially identifiable images or data included in this article.

## Author contributions

CG, SR, and DD’L conceived and designed the study. CG collected the data and wrote the first draft of the manuscript, analyzed the data, and revised the manuscript with contributions from DD’L. All authors contributed to the article and approved the submitted version.

## Funding

This research was not covered by a grant or award. However, the London Borough of Hounslow covered the research costs associated with this project.

## Conflict of interest

SR is employed by the London Borough of Hounslow and has a professional relationship with the setting of interest, however, her role in the study was limited to contributing to the initial research proposal. The research was conducted independently by researchers (CG, DD’L) from University College London.

The remaining authors declare that the research was conducted in the absence of any commercial or financial relationships that could be construed as a potential conflict of interest.

## Publisher’s note

All claims expressed in this article are solely those of the authors and do not necessarily represent those of their affiliated organizations, or those of the publisher, the editors and the reviewers. Any product that may be evaluated in this article, or claim that may be made by its manufacturer, is not guaranteed or endorsed by the publisher.

## Supplementary material

The Supplementary material for this article can be found online at: https://www.frontiersin.org/articles/10.3389/fpubh.2023.1226912/full#supplementary-material
