# Supplementary material for: Anticipated barriers and enablers to signing up for a weight management program after receiving an opportunistic referral from a general practitioner
Source: Front Public Health. 2023 Sep 21;11:1226912. doi: 10.3389/fpubh.2023.1226912 (PMC10552260; doi:10.3389/fpubh.2023.1226912)
Supplement: Supplementary file 2 [file Data_Sheet_2.docx]

**Supplementary file 2**

15-Point Checklist of Criteria for Good Thematic Analysis (Braun & Clarke, 2006).

| Process | No. | Criteria | Response |
| --- | --- | --- | --- |
| Transcription | 1 | The data have been transcribed to an appropriate level of detail, and the transcripts have been checked against the tapes for ‘accuracy’ | Yes, accuracy was checked during the familiarisation and coding stage and data was checked after transcription. |
| Coding | 2 | Each data item has been given equal attention in the coding process | Yes. We reviewed all quotations to generate coding. |
|  | 3 | Themes have not been generated from a few vivid examples (an anecdotal approach), but instead the coding process has been thorough, inclusive and comprehensive | Yes, codes, preliminary candidate themes, and final themes were discussed between the primary researcher and senior researcher. A codebook including illustrative quotes and definitions was developed, and inter-rater reliability was assessed. |
|  | 4 | All relevant extracts for all each theme have been collated | Yes. |
|  | 5 | Themes have been checked against each other and back to the original data set | Yes. |
|  | 6 | Themes are internally coherent, consistent, and distinctive | Yes. |
| Analysis | 7 | Data have been analysed- interpreted, made sense of- rather than just paraphrased or described | Yes. Evident from the results. This was done through thorough discussion between the primary researcher and senior researcher. |
|  | 8 | Analysis and data match each other- the extracts illustrate the analytic claims | Yes. This can be seen in the results table which depicts quotes alongside final themes. |
|  | 9 | Analysis tells a convincing and well organised story about the data and topic | Yes. |
|  | 10 | A good balance between analytical narrative and illustrative extracts is provided | Yes. |
| Overall | 11 | Enough time has been allocated to complete all phases of the analysis adequately, without rushing a phase or giving it a once-over-lightly | Yes. |
| Written report | 12 | The assumptions about, and specific approach to, thematic analysis are clearly explicated | Yes, stated in the methods section. |
|  | 13 | There is good fit between what you claim you do, and what you show you have done- i.e. described method and reported analysis are consistent | Yes, we state that our analyses were grounded in a realism paradigm and post-positivism epistemological approach. The steps in our approach and reasons behind them are outlined clearly. |
|  | 14 | The language and concepts used in the report are consistent with the epistemological position of the analysis | Yes. See #13. |
|  | 15 | The researcher is positioned as *active* in the research process; themes do not just ‘emerge’ | Yes. Themes were developed using the TDF as the central organising concept. |
