# Supplementary material for: Anticipated barriers and enablers to signing up for a weight management program after receiving an opportunistic referral from a general practitioner
Source: Front Public Health. 2023 Sep 21;11:1226912. doi: 10.3389/fpubh.2023.1226912 (PMC10552260; doi:10.3389/fpubh.2023.1226912)
Supplement: Supplementary file 4 [file Table_4.docx]

**Table 4**

Summary of themes, sub-themes, frequencies, and additional illustrative quotes

| Theme  (TDF domain; *n=8*) | Sub-theme (*n=15*) | Frequency (Max *n= 18*) | Barrier / Enabler / Mixed | Illustrative quotes |
| --- | --- | --- | --- | --- |
| 1. Beliefs about consequences | 1.1 Efficacy of the program | 18 | Mixed | “I don't think the doctor saying there’s a 12-week course, go to Isleworth on this date and sign up on this Zoom call on that date is the key” (Female, 45 - 54, White British or other White background, with overweight) |
|  | 1.2 Need for a program | 17 | Mixed | “I think I’m on like 27 BMI at the moment, I’m not sure. But to fix that, I know I can just carry on doing what I was doing before” (Male, 25 - 34, Asian or Asian British, with obesity) |
|  | 1.3 Compatibility with lifestyle | 14 | Mixed | “I work full-time and I have two children. So I couldn’t go, for example, at aerobics at Isleworth at, you know, 10am” (Female, 45 - 54, White British or other White background, with overweight) |
|  | 1.4 Negative emotional response to participation | 4 | Barrier | “You’ll talk to people and they’ll make you feel like you’ve been doing something wrong … And instead, you want… to be able to share honestly without feeling judged” (Female, 25 - 34, White British or other White background, with obesity) |
| 2. Knowledge | 2.1 Having practical information | 18 | Enabler | “… as long as I know, what it involves, and how often, and how long, and where as well, the locations and everything … it shouldn’t be a problem” (Male, 45 - 54, White British or other White background, with overweight) |
|  | 2.2 Misconceptions of who weight management programs are for | 15 | Barrier | “I think it’s more people that either have medical problems or they’ve been diagnosed with pre-diabetes” (Male, 45 - 54, Mixed or multiple ethnic groups, with overweight) |
|  | 2.3 Understanding benefits of the program | 12 | Enabler | “Yeah, I think it would be I suppose what would it add to my kind of health routine generally, like is it providing something that I’m missing?” (Male, 25 - 34, White British or other White background, with overweight) |
| 3. Goals | 3.1 Importance of weight loss | 18 | Mixed | “Even though I’ve got other priorities, you know, I’m carrying the weight with me, it’s kind of dragging me down on a daily basis so it has to be a priority” (Female, 35 - 44, Asian or Asian British, with obesity) |
| 4. Social influences | 4.1 Trust in GP and their recommendation | 16 | Mixed | “I mean the surgery that I go to, I trust the doctors there. If I didn't feel that way, then I would limit my interactions and move someplace else and wouldn't do this programme” (Female, 25 - 34, Asian or Asian British, with overweight) |
|  | 4.2 Others experience with weight management programs | 11 | Mixed | “I had some friends again about Weightwatchers, and unfortunately, it didn’t work, and the feedback was, how would I say it, well a bit negative” (Male, 45 - 54, White British or other White background, with overweight) |
| 5. Social / professional role and identity | 5.1 Fit for the program | 15 | Mixed | “I wear, I cover, so I probably wouldn’t feel comfortable if there was like other men there … it’s like for modest dressing. With women, it’s OK” (Female, 35 - 44, Asian or Asian British, with obesity) |
| 6. Emotion | 6.1 Negative emotional response to signing up | 12 | Barrier | “I might feel a bit frustrated because I have been making better choices, and I have been more active, and I'm trying to get healthier … I think that's an ego thing” (Female, 25 - 34, Asian or Asian British, with overweight) |
| 7. Environmental context and resources | 7.1 Financial implications of joining a program | 7 | Barrier | “… that’s why I brought up this thing about one’s employer. How are they going to be supporting? Are they going to say “yes, this person is involved in this programme, that’s wonderful and yes, she needs to have that time every Wednesday or every other week”” (Female, 55 - 64, Black, Black British, Caribbean or African, with obesity) |
|  | 7.2 Needing time to think, plan and discuss | 2 | Barrier | "I wouldn’t necessarily sign up in any GP’s office because it’s very hard because that document, it took me five, ten minutes to properly read what it involves” (Male, 35 - 44, Asian or Asian British, with obesity) |
| 8. Beliefs about capability | 8.1 Readiness for change | 7 | Enabler | “I think it needs to be something you need to be in the mindset to do and to then sign up for this … you have to be mentally prepared to make that commitment” (Female, 35 - 44, Asian or Asian British, with obesity) |
