# Supplementary material for: Anticipated barriers and enablers to signing up for a weight management program after receiving an opportunistic referral from a general practitioner
Source: Front Public Health. 2023 Sep 21;11:1226912. doi: 10.3389/fpubh.2023.1226912 (PMC10552260; doi:10.3389/fpubh.2023.1226912)
Supplement: Supplementary file 5 [file Data_Sheet_5.docx]

**Supplementary file 5**

Weight management one pager shared with participants
