# Supplementary material for: Anticipated barriers and enablers to signing up for a weight management program after receiving an opportunistic referral from a general practitioner
Source: Front Public Health. 2023 Sep 21;11:1226912. doi: 10.3389/fpubh.2023.1226912 (PMC10552260; doi:10.3389/fpubh.2023.1226912)
Supplement: Supplementary file 6 [file Data_Sheet_6.docx]

**Supplementary file 6**

Interview guide

| # | **Questions and prompts** | **TDF domain** |
| --- | --- | --- |
|  | **Knowledge and environmental influences** |  |
| 1 | Have you heard about these types of weight management programs before?  *Prompts:*  (if yes) Can you tell me more about that?  (if no) Have you heard of similar types of programs?  (Both) Were you aware that the borough of Hounslow offers these types of programs? | Knowledge |
| 2 | What do you think about the outline of the program that I’ve just explained to you?  *Prompts:*  (Both) Would that have any impact on your interest in signing up? | Environmental Context and Resources |
|  | Can you tell me about the last time you saw your GP?  *Prompts:*  (Both) Do you always see the same GP? |  |
|  | **Decision processes & reinforcement** |  |
| 3 | Can you tell me about the things that you would consider before signing up to the program?  *Prompts:*  (Both) Can you talk me through your thought process?  (Both) What would you think about beforehand? | Memory, Attention and Decision Processes |
| 4 | Is there anything that would make you want to sign up for the program?  *Prompts:*  (Both) Is there anything that would change your mind about that?  (Both) Or anything that could be done to influence your decision? | Reinforcement |
|  | **Social influences, role & identity** |  |
| 5 | Have you ever talked about similar weight management programs with other people?  *Prompts:*  (If yes / no) What sort of programs have you discussed?  (Both) Do you know of anyone who has signed up to these programs before? | Social influences |
|  | What would your family / friends think about you signing up to this program?  *Prompts:*  (If yes / no) And would that influence whether or not you signed up?  (Both) Would you care about what they think?  (Both) Would you seek advice from family / friends before signing up? |  |
|  | If your GP recommended that you sign up to this program, would you take their advice?  *Prompts:*  (Both) Can you tell me more about why you would / wouldn’t take their advice? |  |
| 6 | Compared to others in your neighbourhood, would you say that you’re the type of person who would sign up to this program?  *Prompts:*  (If yes / no) Can you tell me more about that?  (Both) What type of people normally sign up to these programs? | Social / Professional Role and Identity |
|  | **Beliefs about capabilities and skills** |  |
| 7 | Would you feel confident talking to your GP about signing up?  *Prompts:*  (Both) Is there anything that you think would stand in your way?  (Both) How easy or difficult do you think it will be for you? | Belief about Capabilities |
| 8 | Would you feel able to sign up to the program without any issues?  *Prompts:*  (if no) What do you think you would struggle with?  (if yes) Can you tell me more about why you wouldn’t struggle? | Cognitive and interpersonal skills |
| 9 | How optimistic are you that you would be able to sign up to this program?  *Prompts:*  (Both) Can you tell me more about that? | Optimism |
|  | **Emotions, intentions, goals, consequences** |  |
| 10 | How would you feel if your GP recommended this program to you?  *Prompts:*  (Both) Do any emotions come up for you?  (Both) Can you tell me a bit more about that? | Emotion |
| 11 | Compared to your other priorities, do you think signing up to a weight management program is important?  *Prompts:*  (Both) Where does it sit in your list of priorities?  (Both) Is weight management in general a priority for you? | Goals |
| 12 | Have you ever thought about signing up to one of these programs in the past?  *Prompts:*  (If yes) Did you end up signing up for it?  (If no) Why have you not considered signing up?  (Both) Do you plan to participate in this type of program in the future? | Intentions |
| 13 | If your GP recommended that you sign up to the program, are there any external barriers that would make it difficult for you to sign up?  *Prompts:*  (if yes) What sort of barriers do you think you would come across?  (if no) Can you tell me more about why you don’t see any barriers to signing up? | Beliefs about consequences |
|  | What do you think the good and bad things about signing up are?  *Prompts:*  (Both) Can you tell me more about that? |  |
|  | **Wrap up** |  |
| 14 | Thank you very much for your time today. We will follow up with you by email to arrange your voucher as a thank you for taking part. Is there anything else that would affect whether you sign up to the program that we haven’t spoken about today? | *[Wrap up and ending]* |
